# The burden of bacterial skin infection, scabies and atopic dermatitis among urban-living Indigenous children in high-income countries: a protocol for a systematic review

**DOI:** 10.1186/s13643-022-02038-8

**Published:** 2022-08-09

**Authors:** Bernadette M. Ricciardo, Heather-Lynn Kessaris, Sujith Prasad Kumarasinghe, Jonathan R. Carapetis, Asha C. Bowen

**Affiliations:** 1 University of Western Australia, Crawley, Western Australia Australia; 2 Wesfarmers Centre for Vaccines and Infectious Diseases, Telethon Kids Institute, Nedlands, Western Australia Australia; 3 Fiona Stanley Hospital, Murdoch, Western Australia Australia; 4 Royal Darwin Hospital, Tiwi, Northern Territory Australia; 5 Perth Children’s Hospital, Nedlands, Western Australia Australia

**Keywords:** Bacterial skin infection, Impetigo, Scabies, Atopic dermatitis, Eczema, Urban, Indigenous, Children, High-income countries, Epidemiology

## Abstract

**Background:**

Bacterial skin infections and scabies disproportionately affect children in resource-poor countries as well as underprivileged children in high-income countries. Atopic dermatitis is a common childhood dermatosis that predisposes to bacterial skin infection. In Australia, at any one time, almost half of all Aboriginal and Torres Strait Islander children living remotely will have impetigo, and up to one-third will also have scabies. Yet, there is a gap in knowledge of the skin infection burden for urban-living Australian Aboriginal and Torres Strait Islander children, as well as atopic dermatitis which may be a contributing factor. The objective of this study is to provide a global background on the burden of these disorders in Indigenous urban-living children in high-income countries. These countries share a similar history of colonisation, dispossession and subsequent ongoing negative impacts on Indigenous people.

**Methods:**

This protocol follows the Preferred Reporting Items for Systematic Review and Meta-Analyses Protocols statement. Observational studies reporting incidence and/or prevalence data on bacterial skin infection, scabies and/or atopic dermatitis in urban-living Indigenous children in high-income countries will be included. Literature searches will be conducted in several international electronic databases (from 1990 onwards), including MEDLINE, Embase, EmCare, Web of Science and PubMed. Reference lists and citation records of all included articles will be scanned for additional relevant manuscripts. Two investigators will independently perform eligibility assessment of titles, abstract and full-text manuscripts, following which both investigators will independently extract data. Where there is disagreement, the senior author will determine eligibility. The methodological quality of selected studies will be appraised using an appropriate tool. Data will be tabulated and narratively synthesised. We expect there will be insufficient data to perform meta-analysis.

**Discussion:**

This study will identify and evaluate epidemiological data on bacterial skin infection, scabies and atopic dermatitis in urban-living Indigenous children in high-income countries. Where available, the clinical features, risk factors, comorbidities and complications of these common childhood skin disorders will be described. The evidence will highlight the burden of disease in this population, to contribute to global burden of disease estimates and identify gaps in the current literature to provide direction for future research.

**Systematic review registration:**

PROSPERO CRD42021277288

**Supplementary Information:**

The online version contains supplementary material available at 10.1186/s13643-022-02038-8.

## Background

Bacterial skin infections commonly present as impetigo, cellulitis or abscess. They are typically caused by infection with the bacteria *Staphylococcus aureus* (the predominant organism in temperate regions) and/or *Streptococcus pyogenes* (the predominant organism in tropical regions) [1]. They may be the result of direct inoculation of pathogenic bacteria (primary infection), or they may occur at a site of skin injury or inflammation where the skin barrier function is disrupted (secondary infection). Such skin inflammation in children is often the result of a pruritic skin disorder, commonly scabies and atopic dermatitis. Bacterial skin infections are readily visible, adversely affect wellbeing and self-image and contribute to general poor health. Without treatment, they can lead to serious and sometimes fatal sequelae, including invasive infection (i.e. sepsis, osteomyelitis) and post-infectious complications (i.e. acute rheumatic fever, acute post-streptococcal glomerulonephritis) [2–5].

Bacterial skin infections are common in childhood with the global paediatric population suffering from impetigo at any one time estimated to be > 162 million [6]. Based on this systematic review of 89 available studies, including 145,028 children assessed for impetigo between 2000 and 2015, the median childhood prevalence was estimated at 12.3% (interquartile range [IQR] 4.2–19.4%) [6]. While high, limitations to this approach include the sparse number and geographical spread of population that contributed to this review — with only 15 of 89 studies conducted in urban populations, none of whom was indigenous children in high-income countries. The highest burden of impetigo is seen in children from marginalised communities of high-income countries where the median prevalence is 19.4% (*IQR* 3.9–43.3%). Australian Aboriginal children in remote northern Australia have the highest documented prevalence of impetigo worldwide. In this cohort, the median prevalence is 45% (*IQR* 34–49%), equating to almost half of all Aboriginal children in remote Australia with impetigo at any one time [6, 7].

Children with scabies are high risk for secondary bacterial infection with impetigo; as a result, areas with a high prevalence of one condition often also have a high prevalence of the other [1]. It is estimated that up to one-third of all Aboriginal children in remote Australia have scabies at any one time [6, 8], with the reported prevalence ranging from 16.1 to 35% [7].

Atopic dermatitis (eczema) is a risk factor for recurrent and severe bacterial skin infection, most commonly due to *S. aureus* and *S. pyogenes* [9]. Approximately, 1 in 3 Australian infants will develop atopic dermatitis by 12 months, [10] and while it has long been thought that Aboriginal people had a lower prevalence of eczema than their non-Aboriginal peers, a recent study revealed comparable rates between these populations [11].

While bacterial skin infections are well documented and at the highest rate in the world in remote-living Australian Aboriginal children, research is lacking on the burden of skin disease for Aboriginal children living in urban areas. Urban-living Aboriginal children in Australia now comprise over 75% of all Aboriginal children; however, little is known about skin infections in this population [12]. Furthermore, there is a gap in knowledge of the burden of predisposing pruritic conditions, such as scabies and atopic dermatitis, in this cohort. Given this, we seek to investigate the burden of these diseases in urban-living Indigenous children, specifically those in high-income countries that share a similar history of colonisation, dispossession and subsequent ongoing negative impacts on Indigenous people. We will systematically evaluate observational epidemiological studies that present prevalence and/or incidence data for bacterial skin infection, scabies and atopic dermatitis among urban-living Indigenous children in high-income countries.

## Methods

This study protocol has been registered with PROSPERO (registration ID: CRD42021277288) and is developed and will be reported in accordance with the Preferred Reporting Items for Systematic Reviews and Meta-Analyses Protocol (PRISMA-P) statement (Additional file 1) [13–15]. A Preferred Reporting Items for Systematic Reviews and Meta-Analyses literature search extension (PRISMA-S) checklist will be submitted with the final manuscript [16].

### Eligibility criteria

#### Study design

Primary observational studies with epidemiological data on bacterial skin infection, scabies or atopic dermatitis in urban-living Indigenous children in high-income countries will be eligible for inclusion. These may be population-based or institutional-based studies. We will include cross-sectional studies and cohort studies (retrospective and prospective) and exclude case-control studies, case series, case studies, randomised controlled trials, reports, commentaries, letters, editorials, book chapters, conference proceedings, theses and animal studies. We will also exclude review articles and opinion papers; however, these will be used to identify any potential primary observational studies that may have been missed in our search.

#### Study population

The review will include Indigenous children and/or adolescents (≤ 18 years old) of any gender who have been diagnosed with a bacterial skin infection, scabies or atopic dermatitis. While the initial vision for this systematic review was to include *all* dermatological disorders, a preliminary search strategy revealed a paucity of published literature on other infectious (including tinea, pediculosis capitis and Buruli ulcer) and non-infectious dermatological conditions in this population and context; hence, the focus was narrowed.

The participants must be urban-living, and since there is heterogeneity in how urban is defined globally, we will use the definition of “urban” as applied by the country in which the study is set at the time the study was performed. Consistent with the degree of urbanisation classification, we will consider urban areas to include cities, towns and semi-dense areas, and where possible, we will categorise our urban studies using these sub-headings [17].

We anticipate there being studies set in major and regional cities that do not provide a breakdown for urban-living versus rural-living participants. We will contact the corresponding author of these studies to determine if they have unpublished data specific for urban-living Indigenous children, and if so, these data will be requested and included. If specific data on urban-living Indigenous children are not available, these studies will also be included but considered in a separate category of combined urban- and rural-living children treated in an urban hospital.

The study setting is high-income countries, as classified by the Organization for Economic Co-operation and Development (OECD) [18]. We are particularly interested in high-income countries that share a similar history of colonisation with the subsequent displacement of Indigenous peoples, including Australia, New Zealand, the USA, Canada, Denmark, Finland, Greenland, Norway and Sweden.

Countries of all climates will be included. Where climate data is not reported, the Koppen climate classification system will be used to code the climate by country (tropical, arid, temperate, cold and polar) [19].

#### Outcomes

The primary outcome will be the prevalence and/or incidence of bacterial skin infection (including impetigo, cellulitis and abscess), scabies or atopic dermatitis (eczema) in urban-living Indigenous children of high-income countries. We will use author-reported definitions of these diseases.

The secondary outcomes will be the clinical features (phenotype), risk factors, comorbidities and complications of bacterial skin infection, scabies and atopic dermatitis in urban-living Indigenous children of high-income countries. The microbiology of the bacterial skin infections in this population will also be reported.

### Information sources

Literature searches will be conducted in several international electronic databases including MEDLINE (Ovid), Embase (Ovid), EmCare (Ovid), Web of Science and PubMed.

The reference lists of all included full-text articles and relevant review articles will be scanned for additional manuscripts for inclusion. Citation searching of all included full-text articles will also be performed.

### Search strategy

The literature searches will be designed and conducted by the review team with the help of a health information specialist. The search strategy will be conducted taking into account the “CoCoPop” (condition, context and population) framework [20]. It will use a combination of keywords and subject headings relating to the population and outcomes of interest. A sample search strategy for MEDLINE (Ovid) search strategy is included (Additional file 2). The search will be restricted to studies published since 1990. Any study published in English will be considered for inclusion in the review.

### Study records

#### Data management

The search results will be uploaded to EndNote reference management software where titles and abstracts will be screened. Full-text articles for assessment against the inclusion criteria will be collated in a Microsoft Excel spreadsheet. Extracted data from included articles will also be recorded in a Microsoft Excel spreadsheet.

#### Selection process

Two authors will independently perform the search strategy, removing duplicates and screening the titles and abstracts for relevance. Following this, the full-text articles will be retrieved and assessed against the inclusion criteria. Any disagreement between the two reviewers regarding a potential study inclusion will be resolved through discussion with the senior author as third reviewer if needed. In the case that data is duplicated in more than one study, the study with the most comprehensive data will be included. The literature selection and the reasons for study exclusion will be recorded in a Microsoft Excel spreadsheet and then documented in a PRISMA flow diagram.

For included full-text articles and relevant reviews, the reference lists and citations will be scanned to identify additional studies that may have been missed in the initial search.

The included manuscripts will be divided in to the following conditions — bacterial skin infection, scabies and atopic dermatitis.

#### Data collection process

A data extraction form will be prepared to capture information on study characteristics, participants and study outcomes. Two authors will independently extract the data. Any disagreement between the two reviewers as relates to data extraction will be resolved through discussion with a third reviewer if needed.

#### Data items

Extracted data will include the following:

- Publication details: publication year, first author, title, journal and source of funding
- Design details: type of study, aim(s) of study, inclusion criteria, exclusion criteria, diagnostic criteria (for bacterial skin infection, scabies and atopic dermatitis), qualification(s) of person conducting the screening, method of data collection, recruitment and sampling methods, number of participants and response rate
- Study setting details: study site (school, juvenile custodial centre, primary health-care service, hospital emergency department, hospital inpatient), town/city where study conducted, population of town/city where study conducted at the time the study was performed, country where study conducted, definition of “urban” in country where study conducted at the time the study was performed, degree of urbanization (city, town, semi-dense area) and climate of town/city where study conducted (tropical, arid, temperate, cold and polar)
- Study participant details: age, sex and ethnicity
- Data for primary outcome measures: all reported estimates or sufficient information to calculate an estimate of the point prevalence, period prevalence, cumulative incidence and/or incidence rates of bacterial skin infection, scabies and atopic dermatitis
- Data for secondary outcome measures: clinical features (phenotype), risk factors, comorbidities and complications of bacterial skin infection, scabies and atopic dermatitis, culture-proven aetiology of bacterial skin infection
- Limitations: selection bias, response bias, information bias, limitations of assessment tool(s) used and limitations reported by study authors.

#### Risk-of-bias assessment

Two authors will independently appraise the studies. The assessment of methodological quality will be based on the Joanna Briggs Institute (JBI) appraisal checklist for studies reporting prevalence data [21]. Risk-of-bias assessments will subsequently be rated as high, low or unclear in a “risk-of-bias” table. Any disagreements that arise will be resolved via a consensus decision of the review team members.

### Data synthesis

The results for each condition (i.e. bacterial skin infection, scabies, atopic dermatitis) will be presented separately. Quantitative and qualitative findings for each condition will be described in narrative form and, where appropriate, synthesised into tables and figures to aid in data interpretation. A single, comprehensive set of synthesised quantitative findings will be presented in a “Summary of Findings” table for each condition.

If appropriate, and if such data can be retrieved from the manuscripts, subgroup analysis (with median prevalence) may be carried out using the following:

- Age groups: 0–1 years, 1–5 years, 6–10 years and > 11 years
- Sex: male and female
- Continental population groups: the Oceanic Ancestry Group, American Native Continental Ancestry Group and European Continental Ancestry Group
- Degree of urbanisation: city, town and semi-dense area
- Climate: tropical, arid, temperate, cold and polar

We expect there to be insufficient data to conduct meta-analysis.

### Meta-biases

Studies will be included only once to reduce the risk of publication bias. Selective outcome reporting will be considered when articles describe the outcomes in “Methods” but fail to report them in “Results.”

## Discussion

This systematic review will identify and report the estimated prevalence and/or incidence of bacterial skin infections, scabies and atopic dermatitis among urban-living Indigenous children of high-income countries. This has not been previously collated in the published literature. Where possible, subgroup analysis will be performed based on age, sex, continental population group, degree of urbanisation and climate, and we will describe the clinical features, risk factors, comorbidities and complications of these common childhood skin disorders. The expected high heterogeneity between studies will likely preclude meta-analysis. Limitations of the studies will be discussed in detail.

To our knowledge, this will be the first review to systematically synthesise and collate the available epidemiological evidence on bacterial skin infections, scabies and atopic dermatitis in urban-living Indigenous children of high-income countries. The results will describe the burden of these skin conditions, highlighting the risk factors and comorbidities and identifying gaps in the literature to direct future research. In addition, this will be an important contribution to the global burden of disease estimates as these populations have not previously been included. Implications of the review and suggestions for future research will be provided. The resulting manuscript will be submitted for publication.

## Supplementary Information

**Additional file 1.** PRISMA-P 2015 Checklist: recommended items to address in a systematic review.

**Additional file 2.** MEDLINE (Ovid) search strategy.

## Data Availability

Not applicable

## Abbreviations

IQR: Interquartile range
JBI: Joanna Briggs Institute
OECD: Organization for Economic Co-operation and Development
PRISMA: Preferred Reporting Items for Systematic Reviews and Meta-analyses
PRISMA-P: Preferred Reporting Items for Systematic Reviews and Meta-Analyses Protocol
PRISMA-S: Preferred Reporting Items for Systematic Reviews and Meta-Analyses literature search extension
*S. aureus*: *Staphylococcus aureus*
*S. pyogenes*: *Streptococcus pyogenes*
