# Supplementary material for: The burden of bacterial skin infection, scabies and atopic dermatitis among urban-living Indigenous children in high-income countries: a protocol for a systematic review
Source: Syst Rev. 2022 Aug 9;11:159. doi: 10.1186/s13643-022-02038-8 (PMC9361683; doi:10.1186/s13643-022-02038-8)
Supplement: Supplementary file 1 — Additional file 1. PRISMA-P 2015 Checklist: recommended items to address in a systematic review. [file 13643_2022_2038_MOESM1_ESM.docx]

**Additional File 1 – PRISMA-P 2015 Checklist: recommended items to address in a systematic review**

| Section and topic | Item No | Checklist item | Signpost |
| --- | --- | --- | --- |
| ADMINISTRATIVE INFORMATION | | |  |
| Title: |  |  |  |
| Identification | 1a | Identify the report as a protocol of a systematic review | The burden of bacterial skin infection, scabies and atopic dermatitis among urban-living Indigenous children in high-income countries: a protocol for a systematic review. |
| Update | 1b | If the protocol is for an update of a previous systematic review, identify as such | N/A |
| Registration | 2 | If registered, provide the name of the registry (such as PROSPERO) and registration number | PROSPERO registration number: submitted 06/09/2021 |
| Authors: |  |  |  |
| Contact | 3a | Provide name, institutional affiliation, e-mail address of all protocol authors; provide physical mailing address of corresponding author | **Dr Bernadette Ricciardo (Corresponding author)**  Telethon Kids Institute  University of Western Australia  Derbarl Yerrigan Health Service  Fiona Stanley Hospital.  Royal Perth Hospital.  Email: [bernadette.ricciardo@health.wa.gov.au](mailto:bernadette.ricciardo@health.wa.gov.au)  **Address**: Telethon Kids Institute. PO Box 855, West Perth, WA, 6872.    Dr Heather-Lynn Kessaris  Royal Perth Hospital  [heatherlynnkessaris@gmail.com](mailto:heatherlynnkessaris@gmail.com)  Clin/Prof Sujith Prasad Kumarasinghe  University of Western Australia  [prasadkumarasinghe@yahoo.com](mailto:prasadkumarasinghe@yahoo.com)  Prof Jonathan Carapetis  University of Western Australia  Telethon Kids Institute  [Jonathan.Carapetis@telethonkids.org.au](mailto:Jonathan.Carapetis@telethonkids.org.au)  A/Prof Asha Bowen  University of Western Australia  Telethon Kids Institute  Perth Children’s Hospital  [asha.bowen@health.wa.gov.au](mailto:asha.bowen@health.wa.gov.au) |
| Contributions | 3b | Describe contributions of protocol authors and identify the guarantor of the review | BR wrote the protocol. AB conceived the initial idea for the review, which was refined by BR. HK, PK, JC and AB critically appraised the protocol and contributed to its design and development. All authors approve the final version and take responsibility for its content. |
| Amendments | 4 | If the protocol represents an amendment of a previously completed or published protocol, identify as such and list changes; otherwise, state plan for documenting important protocol amendments | N/A |
| Support: |  |  |  |
| Sources | 5a | Indicate sources of financial or other support for the review | The authors have not received any external funding |
| Sponsor | 5b | Provide name for the review funder and/or sponsor | N/A |
| Role of sponsor or funder | 5c | Describe roles of funder(s), sponsor(s), and/or institution(s), if any, in developing the protocol | N/A |
| INTRODUCTION | | |  |
| Rationale | 6 | Describe the rationale for the review in the context of what is already known | Background |
| Objectives | 7 | Provide an explicit statement of the question(s) the review will address with reference to participants, interventions, comparators, and outcomes (PICO) | The objective of the review |
| METHODS | | |  |
| Eligibility criteria | 8 | Specify the study characteristics (such as PICO, study design, setting, time frame) and report characteristics (such as years considered, language, publication status) to be used as criteria for eligibility for the review | Methods |
| Information sources | 9 | Describe all intended information sources (such as electronic databases, contact with study authors, trial registers or other grey literature sources) with planned dates of coverage | Information sources |
| Search strategy | 10 | Present draft of search strategy to be used for at least one electronic database, including planned limits, such that it could be repeated | See Additional file 2 |
| Study records: |  |  |  |
| Data management | 11a | Describe the mechanism(s) that will be used to manage records and data throughout the review | Data management |
| Selection process | 11b | State the process that will be used for selecting studies (such as two independent reviewers) through each phase of the review (that is, screening, eligibility and inclusion in meta-analysis) | Selection process |
| Data collection process | 11c | Describe planned method of extracting data from reports (such as piloting forms, done independently, in duplicate), any processes for obtaining and confirming data from investigators | Data collection process |
| Data items | 12 | List and define all variables for which data will be sought (such as PICO items, funding sources), any pre-planned data assumptions and simplifications | Data items |
| Outcomes and prioritization | 13 | List and define all outcomes for which data will be sought, including prioritization of main and additional outcomes, with rationale | Outcomes |
| Risk of bias in individual studies | 14 | Describe anticipated methods for assessing risk of bias of individual studies, including whether this will be done at the outcome or study level, or both; state how this information will be used in data synthesis | Risk of bias assessment |
| Data synthesis | 15a | Describe criteria under which study data will be quantitatively synthesised | Data synthesis |
|  | 15b | If data are appropriate for quantitative synthesis, describe planned summary measures, methods of handling data and methods of combining data from studies, including any planned exploration of consistency (such as I^2^, Kendall’s τ) | N/A (as not performing meta-analysis) |
|  | 15c | Describe any proposed additional analyses (such as sensitivity or subgroup analyses, meta-regression) | Data synthesis |
|  | 15d | If quantitative synthesis is not appropriate, describe the type of summary planned | Data synthesis |
| Meta-bias(es) | 16 | Specify any planned assessment of meta-bias(es) (such as publication bias across studies, selective reporting within studies) | Meta-biases |
| Confidence in cumulative evidence | 17 | Describe how the strength of the body of evidence will be assessed (such as GRADE) | N/A |

*From: Shamseer L, Moher D, Clarke M, Ghersi D, Liberati A, Petticrew M, Shekelle P, Stewart L, PRISMA-P Group. Preferred reporting items for systematic review and meta-analysis protocols (PRISMA-P) 2015: elaboration and explanation. BMJ. 2015;349:g7647.*
