# Supplementary material for: The burden of bacterial skin infection, scabies and atopic dermatitis among urban-living Indigenous children in high-income countries: a protocol for a systematic review
Source: Syst Rev. 2022 Aug 9;11:159. doi: 10.1186/s13643-022-02038-8 (PMC9361683; doi:10.1186/s13643-022-02038-8)
Supplement: Supplementary file 2 — Additional file 2. MEDLINE (Ovid) search strategy. [file 13643_2022_2038_MOESM2_ESM.docx]

**Additional File 2 – MEDLINE (Ovid) search strategy**

| **Search** | **Query** |
| --- | --- |
| #1 | exp american native continental ancestry group/ or exp european continental ancestry group/ or exp oceanic ancestry group/ |
| #2 | (Ethnic* or Aboriginal* or Indigenous* or First Nation* or Native* or First Australian* or Torres Strait Islander* or Maori* or Pacific Islander* or Native Hawaiian* or American Indian* or First American* or Amerind* or Alaska Native* or Eskimo* or Inuit* or Sami*).mp. [mp=title, abstract, original title, name of substance word, subject heading word, floating sub-heading word, keyword heading word, organism supplementary concept word, protocol supplementary concept word, rare disease supplementary concept word, unique identifier, synonyms] |
| #3 | 1 or 2 |
| #4 | Child.mp. or exp Child/ |
| #5 | exp Infant Health/ or Infant/ or exp Infant, Newborn/ or Infant.mp. or exp Infant, Newborn, Diseases/ |
| #6 | exp Adolescent Health Services/ or Adolescent/ or exp Adolescent Health/ or exp Adolescent Medicine/ or adolescent.mp. or exp Adolescent, Hospitalized/ |
| #7 | (School age or preteen* or pre-teen* or teen* or adolescence or youth* or young people* or young adult* or newborn* or neonate* or infancy or children or preschool* or pre-school*).mp. [mp=title, abstract, original title, name of substance word, subject heading word, floating sub-heading word, keyword heading word, organism supplementary concept word, protocol supplementary concept word, rare disease supplementary concept word, unique identifier, synonyms] |
| #8 | 4 or 5 or 6 or 7 |
| #9 | exp Skin Diseases, Eczematous/ or exp Staphylococcal Skin Infections/ or exp Skin Diseases, Bacterial/ or exp Skin Diseases, Infectious/ |
| #10 | exp Staphylococcus aureus/ |
| #11 | exp Streptococcus pyogenes/ |
| #12 | impetigo.mp. or exp Impetigo/ |
| #13 | pyoderma.mp. or exp Pyoderma/ |
| #14 | exp Cellulitis/ or cellulitis.mp. |
| #15 | scabies.mp. or exp Scabies/ |
| #16 | exp Sarcoptes scabiei/ or sarcoptes scabies.mp. |
| #17 | exp Eczema/ or eczema.mp. |
| #18 | exp Dermatitis, Atopic/ or dermatitis.mp. |
| #19 | (skin infection* or bacterial skin infection* or echthyma or folliculitis or furunculosis or abscess* or erysipelas or atopic eczema or endogenous eczema or atopic dermatitis).mp. [mp=title, abstract, original title, name of substance word, subject heading word, floating sub-heading word, keyword heading word, organism supplementary concept word, protocol supplementary concept word, rare disease supplementary concept word, unique identifier, synonyms] |
| #20 | 9 or 10 or 11 or 12 or 13 or 14 or 15 or 16 or 17 or 18 or 19 |
| #21 | (Incidence or Prevalence or Epidemiology or Burden).mp. [mp=title, abstract, original title, name of substance word, subject heading word, floating sub-heading word, keyword heading word, organism supplementary concept word, protocol supplementary concept word, rare disease supplementary concept word, unique identifier, synonyms] |
| #22 | 3 and 8 and 20 and 21 |
| #23 | limit 22 to yr="1990 -Current" |
